# Evaluating a Mobile-Health-Enhanced Behavioral Lifestyle Intervention for Frailty in Diabetes: A Feasibility Study

**DOI:** 10.1093/geroni/igaa057.1292

**Published:** 2020-12-16

**Authors:** Rozmin Jiwani, Jing Wang, Chengdong Li, Sara Espinoza

**Affiliations:** University of Texas Health Science Center at San Antonio, San Antonio, Texas, United States

## Abstract

Type 2 diabetes (T2D) and overweight are significant predictors of frailty in older adults. There are no widely accepted intervention strategies for frailty. The Look AHEAD (Action for Health in Diabetes) trial demonstrated the efficacy of behavioral lifestyle intervention on weight loss and glycemic control in overweight older adults with T2D. It is unknown whether this intervention can prevent/delay frailty in older adults. We designed a feasibility study examining the effect of a behavioral lifestyle intervention enhanced with mobile technology (Fitbit) for self-monitoring of diet and physical activity on frailty and T2D outcomes over 6 months in overweight older adults diagnosed with T2D. Forty older adults were randomized to receive either 10 group sessions vs. one condensed session plus monthly phone calls for 6 months. In this analysis, we are reporting on Fitbit wear adherence and weight changes on the 20 participants in the group session for the first 6 group sessions. The study sample was aged 72.3±6.4 years; 62% female; 52% Hispanic; BMI 33.7±5.9 kg/m2; hemoglobin A1c 7.2%; frailty score 1.1±1.0 kg/m2. Thirteen (65%) are pre-frail, 6 (30%) are non-frail, and 1 (5%) is frail (using Fried criteria). Their weight (lbs.) changed from session 1 (210.2±42.5) to session 6 (196.8±44.2). Ten participants wore Fitbit every day between sessions, averaged at 92±12%. The preliminary evaluation showed the feasibility of using Fitbit to promote self-monitoring adherence in a behavioral lifestyle intervention and a positive trend for weight loss. Evaluating intervention effect on frailty at 6 months will provide us further insights.

